# Zintl Phases as Reactive Precursors for Synthesis of Novel Silicon and Germanium-Based Materials

**DOI:** 10.3390/ma12071139

**Published:** 2019-04-08

**Authors:** Matt Beekman, Susan M. Kauzlarich, Luke Doherty, George S. Nolas

**Affiliations:** 1 Department of Physics, California Polytechnic State University, San Luis Obispo, CA 93407, USA; ladohert@calpoly.edu; 2 Department of Chemistry, University of California, Davis, CA 95616, USA; smkauzlarich@ucdavis.edu; 3 Department of Materials Engineering, California Polytechnic State University, San Luis Obispo, CA 93407, USA; 4 Department of Physics, University of South Florida, Tampa, FL 33620, USA; gnolas@usf.edu

**Keywords:** silicon, germanium, Zintl phase, metastable, allotropes, soft chemistry, nanoparticles, mesostructured materials

## Abstract

Recent experimental and theoretical work has demonstrated significant potential to tune the properties of silicon and germanium by adjusting the mesostructure, nanostructure, and/or crystalline structure of these group 14 elements. Despite the promise to achieve enhanced functionality with these already technologically important elements, a significant challenge lies in the identification of effective synthetic approaches that can access metastable silicon and germanium-based extended solids with a particular crystal structure or specific nano/meso-structured features. In this context, the class of intermetallic compounds known as Zintl phases has provided a platform for discovery of novel silicon and germanium-based materials. This review highlights some of the ways in which silicon and germanium-based Zintl phases have been utilized as precursors in innovative approaches to synthesize new crystalline modifications, nanoparticles, nanosheets, and mesostructured and nanoporous extended solids with properties that can be very different from the ground states of the elements.

## 1. Introduction

For nearly 100 years, the elements silicon and germanium have received much attention from chemists, physicists, and materials scientists due in large part to their immense technological importance in semiconductor and optoelectronic applications as well as their richness in chemical properties [1,2,3]. Although current technologies are dominated by the well-studied (and relatively well understood) diamond structured forms known as *α*-Si and *α*-Ge, there also exist more “exotic” forms of these elements [4], including alternative crystalline modifications [5,6,7,8,9], amorphous states [10], and various nanostructured forms [11,12] that can have remarkably different properties. Early indications of the promise for tuning properties in these elements through structural modification were revealed by studies of porous silicon [13] and expanded low-density crystalline silicon allotropes [14], both of which show increased electronic band gaps relative to *α*-Si. Porous silicon provides a striking example of the influence of size/nanostructure on properties (through the mechanism of quantum confinement [15]), whereas silicon clathrate allotropes demonstrate the importance of atomic arrangement on the electronic structure of a crystal. Since such attributes can be used independently or in combination to obtain materials with new and possibly improved functionality, novel silicon and germanium-based materials comprise more than mere scientific curiosities.

More recently, advanced computational approaches and revived theoretical interest in elemental modifications have established that the possible phase space for new forms of silicon and germanium is much richer than originally thought [16,17,18,19,20]. For example, hundreds of relatively low-energy crystalline modifications of silicon have been theoretically predicted, many of which could have promising light absorption properties, which are of interest for improved photovoltaics [17,18]. The expanded frameworks found in clathrate modifications possess interesting thermal properties, such as tunable thermal expansion [21] and low thermal conductivity [22], and could find application as anodes in lithium ion batteries [23,24]. The growing ability to prepare mesostructured and nanostructured silicon and germanium-based materials provides additional mechanisms to target specific material properties [25]. The key challenge limiting progress in all cases, however, is effective synthetic approaches to experimentally obtain a specific material of interest. For example, although hundreds of crystalline elemental modifications of Si and Ge are conceivable, only a handful of these materials have been prepared in the lab [5]. By definition, all forms other than crystalline *α*-Si and *α*-Ge are thermodynamically metastable under ambient conditions, and such materials are typically difficult to obtain by conventional synthetic approaches.

The use of precursors is particularly attractive for the preparation of metastable and difficult to synthesize solids. Chemical precursors can have structural features that are similar to the desired product, lowering activation energies for transformation and facilitating topotactic reactions. Reducing the need for extensive solid-state diffusion and/or the breaking of strong chemical bonds allows reactions to proceed at lower temperatures. Metastable products can thus be kinetically trapped at local energy minima by halting the reaction at an intermediate step along the path to the most thermodynamically stable products. Different reaction conditions can drive the transformation of the same precursor to different products. In this contribution, we highlight the important role the class of materials known as Zintl phases has assumed in the preparation of new silicon and germanium-based materials. The synthetic approaches discussed below have yielded a variety of new elemental modifications as well as mesostructured solids and nanostructured materials, and hold significant promise for new materials discovery.

## 2. Zintl Ions and Zintl Phases

While there are many design strategies to making new compounds and materials, a very powerful guideline is the Zintl electron counting or Zintl-Klemm concept [26,27,28]. Zintl phases, sometimes referred to as the valence precise intermetallics, are a large and continually growing collection of compounds whose structure and bonding can be rationalized by simple electron counting rules. A number of excellent reviews and texts covering the structure, chemistry, and properties of Zintl phases are available [26,27,28,29,30,31,32], thus we only briefly highlight some important features relevant to their reactivity here.

**Figure 1:**
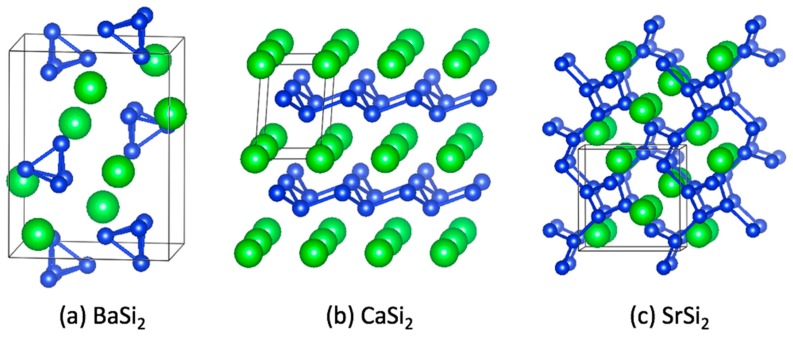
Examples of anionic Zintl subunits found in the crystal structures of three binary ASi_2_ Zintl phases (A = Ca, Ba, Sr). (**a**) BaSi_2_ contains isolated [Si_4_]^4−^ clusters that are charge balanced by Ba^2+^ cations. (**b**) CaSi_2_ contains 2D [Si2]2−∞2 layers that are charge balanced by Ca^2+^ cations. (**c**) SrSi_2_ contains a 3D [Si2]2−∞3 network that is charge balanced by Sr^2+^ cations. In all three of these examples of alkaline earth disilicides, each Si atom is 3-bonded to other Si atoms with a formal charge of −1 per Si atom. (Silicon atoms are shown in blue, alkaline-earth atoms are shown in green.)

Named after the pioneering chemist Eduard Zintl [28,29], binary Zintl phases originally garnered fundamental interest both for their interesting structures and their solution reactivity. The structures of Zintl phases typically feature negatively charged subunits that range from isolated 0D anions or polyatomic clusters to charged 3D networks (Figure 1), such that covalent bonding within the subunits is enabled by transfer of electrons from alkali, alkaline-earth, or rare earth atoms. This charge transfer enables the organization of the constituents in the anionic subunits, which are collectively termed “Zintl ions”, in ways that often mimic structures found in the neighboring elements in the periodic table, and the resulting materials are often (but not always) semiconductors prepared from all metallic elements. While representative classical Zintl phases typically contained *p*-block elements, the Zintl concept has since been expanded to transition metal-containing semiconductors, which show unique combinations of properties, such as colossal magnetoresistance and ferromagnetic semiconductors [33,34]. Zintl phases are also receiving significant attention as potential thermoelectric materials [35,36,37].

There are several features of Zintl phases that lend themselves to use as precursors in materials synthesis. Even from the perspective of traditional high-temperature solid-state reactions, precursors have clear advantages. The elements in a precursor are already homogenously mixed at the atomic level, requiring less solid-state diffusion upon further reaction. The vapor pressure over a compound containing a volatile element, e.g., Na or Hg, is typically less than that over the element so that reactions employing the compound can be easier to control. However, it is the particular crystal chemistry of Zintl phases that make them amenable to use in low temperature and soft chemical reactions that can yield new materials not accessible by conventional approaches. In many cases, the structural subunits in Zintl phases lend themselves to topotactic reactions in which a metastable intermediate (the target product) has a crystallographic structural relationship to the precursor. Also, upon dissolution of these salt-like solids, the polyatomic Zintl ions found in the crystal are often retained in solution and can be subsequently employed as reactive precursors to new materials [27,28,38]. Indeed, Zintl himself studied both the solid-state and the solution chemistry aspect of the compounds that now bear his name [23,38]. It has been demonstrated that oxidative coupling of isolated Zintl clusters can form various 1D polymeric chains [39,40]. The anionic subunits of Zintl phases, including polyanionic clusters (Figure 1a), infinite 2D sheets (Figure 1b), and 3D networks (Figure 1c), essentially comprise covalently bonded structural moieties in the precursor that can act as molecular building blocks from which novel 3D materials can be built or derived via oxidation [41]. Notably, chemical substitutions on various atomic sites in the Zintl ions in the precursor are possible [42,43], enabling a mechanism for adjusting composition and doping of the products derived from them.

## 3. Synthesis of Zintl Phases

The synthesis of most Zintl phases can be achieved via conventional solid-state preparation routes for intermetallic compounds [44]. Many Zintl phases, in particular those that have been used as precursors for synthesis of silicon and germanium-based materials, are thermodynamically stable compounds and can thus be prepared as single phase polycrystalline or single crystalline products by direct reaction of stoichiometric mixtures of the high purity elements in sealed vessels under inert atmosphere. In many cases, both the reactants and the resulting Zintl phases are highly reactive toward moisture and oxygen, thus all handing, including chemical and structural characterization, must be done under a protected environment. Reactions involving alkali metals are typically carried out in welded tantalum or niobium containers, though formation of Nb or Ta silicides in side reactions can occur at higher temperatures [45]. Alternatively, binary Zintl phases can also be prepared in the lab by low temperature reactions with metal hydrides, allowing access to some Zintl phases without the need for niobium or tantalum containers [46]. Binary Zintl phases can also be used as precursors for preparation of ternary or alloyed Zintl phases [47].

## 4. Preparation of New Crystalline Elemental Modifications and Frameworks

It is a rather remarkable fact that a very large number of distinct elemental crystalline structures, perhaps in the tens of thousands, can accommodate Si and Ge atoms in the four-coordinated bonding geometry that these elements prefer, indicating the free energy landscape for these elements is quite complex [4,5,6,7,8,9,14,17,18,19,20]. The deviations in bond lengths and angles for many of these structures (relative to the respective *α*-phase) are sufficiently small, thus the corresponding free energy is sufficiently low, and therefore they should be experimentally accessible [4,5,6,7,8,9,14,17,18,19,20]. Once formed, the strong covalent interactions in these materials provide sufficient energy barriers to the transformation to the ground state (*α*-phase) such that the metastable phase can be kinetically trapped. However, despite average bond distances and angles that are often comparable to those found in the *α*-phases, the variation in crystal structure in the different modifications of silicon and germanium results in widely varying electronic and thermal properties [4,5,6,7,8,9,14,17,18,19,20,21,22], providing unique opportunities to obtain new materials with improved or distinct functionality. Such materials, if synthesized, could potentially be processed into devices with higher performance using many of the mature processing and fabrication technologies already established for silicon and germanium.

It is well known that metastable modifications of many of the elements can be retained upon decompression from high pressure to ambient conditions [48]. This includes several crystalline allotropes of silicon and germanium [7,20,49,50,51]. While such experiments play a critical role in understanding the chemistry and physics of the elements, the products in these cases are typically limited to those that are thermodynamically stable at high pressure. In addition, low-density allotropes, expected to have interesting and perhaps useful properties [6], cannot be accessed by solely adjusting the temperature and/or pressure of the pure elements. On the other hand, chemical methods have the potential to access a richer variety of metastable materials, and Zintl phases are playing a central role as precursors in such preparation routes. The soft chemistry and/or low temperature reactions involving Zintl precursors discussed in this section are particularly promising for the synthesis of new metastable silicon and germanium-based allotropes and frameworks and, as described in the sections that follow, continue to be developed as such.

### 4.1. Chemical Oxidation of Zintl Precursors

**Figure 2:**
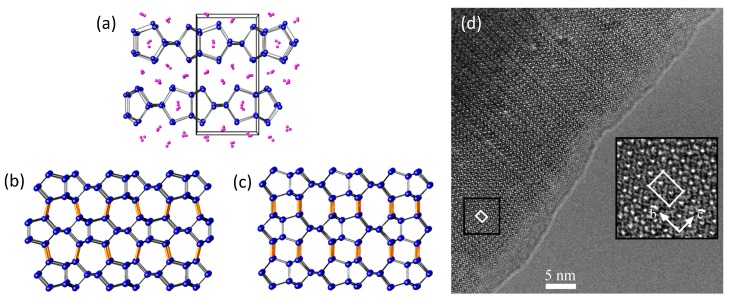
(**a**) Crystal structure of Li_7_Ge_12_ (Li atoms shown in pink, Ge atoms shown in blue); (**b**) and (**c**) show two distinct inter layer couplings that can occur upon oxidation of the [Ge12]7−∞2 layers in Li_7_Ge_12_; (**d**) the structure of *allo*-Ge can be modeled as a statistical mixture of the stackings shown in (**b**) and (**c**), evidenced by this high-resolution transmission electron microscope image showing extensive stacking fault disorder along the c direction; (**a**) through (**c**) reproduced with permission from Zaikina, J.V. et al. (Copyright, 2014, American Chemical Society); (**d**) reproduced with permission from Kiefer, F. et al. (Copyright, 2011, American Chemical Society).

Two of the earliest examples of silicon and germanium allotropes prepared by chemical methods using a Zintl phase precursor were the reports of the so-called *allo*-Ge and *allo*-Si phases [52,53]. Von Schnering et al. reported that these novel modifications could be prepared by deintercalation of Li (and Na) from the layered Zintl phases Li_7_Ge_12_ (Figure 2a) and Li_3_NaSi_6_, respectively, via reaction with benzophenone at ambient temperature [52,53], e.g., according to the reaction (Li_7_Ge_12_ + 7Ph_2_CO → 12*allo*-Ge + 7Li(Ph_2_CO)). In the case of *allo*-Ge, a rough model of the crystal structure was proposed based on interconnection of the Ge layers found in Li_7_Ge_12_ [52]. Potential structural models were subsequently proposed by other workers [54,55], but the structures of *allo*-Si and *allo*-Ge were not definitively solved and remained unknown for several decades. The synthesis and characterization of these materials was recently reinvestigated, and more detailed structural characterization was performed [47,56], supporting the conclusion that the formation of *allo*-Ge proceeds via oxidative coupling of the [Ge12]7−∞2 layers that are present in Li_7_Ge_12_. On the other hand, Zeilinger et al. reported that the preparation of *allo*-Si could not be reproduced with the products from the reaction of Li_15_Si_4_ and Li_3_NaSi_6_ with various protic solvents comprised of mixtures of amorphous Si, nanocrystalline *α*-Si, and clathrate impurities [47]. In both cases, the products of the reactions are microcrystalline powders.

The reinvestigations of the crystal structure of *allo*-Ge confirm that the five-ring topology present in the [Ge12]7−∞2 layers of Li_7_Ge_12_ [57] is retained in *allo*-Ge [56,58], illustrating the efficacy of topotactic reactions employing Zintl phase precursors by oxidation of lone pair electrons in the layers, and the corresponding formation of additional Ge–Ge interlayer and intralayer bonds to convert the 2D layers into a 3D network. However, good agreement with experimental powder X-ray diffraction data could only be achieved by including stacking disorder in the structural model [56]. The presence of extensive stacking disorder in *allo*-Ge can be understood in terms of the different energetically feasible ways in which two layers can couple via translations relative to one another during the process of delithiation and oxidation of the anionic layers (see Figure 2b,c). This suggests that the rapidly proceeding reaction produces stacking faults as Li is removed from the Li_7_Ge_12_ structure [56,58], supporting the idea that the [Ge12]7−∞2 layers in the precursor can couple in various ways [54] depending on the oxidation conditions. When different couplings are possible, such processes can be difficult to control. However, the possibility of coupling the [Ge12]7−∞2 layers in Li_7_Ge_12_ in various different stacking sequences also affords the preparation of distinct allotropes from a single precursor. The recently reported Ge(*oP*32), which can be derived from the structural variants on which the structure of the disordered *allo*-Ge is based (Figure 2b), was prepared by oxidation of Li_7_Ge_12_ in the ionic liquids dodecyltrimethyl-ammonium aluminum tetrachloride (DTAC) or hexyltrimethylammonium aluminum tetrabromide (HTMAB) at 135–145 °C for three to seven days [59]. Interestingly, extensive stacking disorder was not reported in this case, indicating different experimental conditions can direct the transformation of the same precursor into different crystalline products.

**Figure 3:**
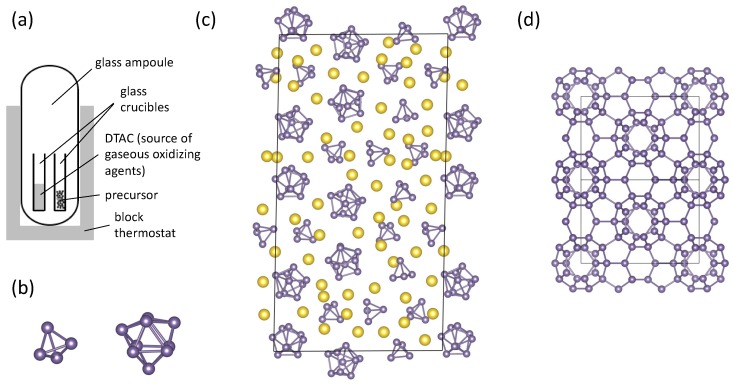
(**a**) Schematic of the reaction vessel used in the synthesis of Ge(*cF*136) (adapted from [63]). (**b**) Isolated [Ge_4_]^4−^ and [Ge_9_]^4−^ Zintl ions found in Na_12_Ge_17_. (**c**) Partial crystal structure of Na_12_Ge_17_ with Na atoms shown in yellow [64]. (**d**) Crystal structure the crystalline Ge modification Ge(*cF*136) that is prepared by mild oxidation of Na_12_Ge_17_ [60,63].

The oxidation of Li_7_Ge_12_ to form Ge allotropes such as the *allo*-Ge or Ge(*oP*32) modifications by linking of the [Ge12]7−∞2 layers in the precursor provides particularly elegant examples of how the charged 2D structural units afforded in layered Zintl precursors can be used to direct the formation of an elemental allotrope by soft chemical methods. However, other Zintl precursors with isolated cluster anions have also been successfully utilized to prepare new crystalline modifications. A striking example is the preparation of Ge(*cF*136) with the cage-like clathrate-II crystal structure (Figure 3d) [60]. The silicon and germanium frameworks in clathrate structures are built from various face-sharing polyhedra, which usually encapsulate guest atoms inside of the framework cages [61,62]. In contrast, a guest-free Ge_136_ framework can also be obtained, constituting a new crystalline Ge allotrope [9,62]. Ge(*cF*136) can be prepared under various conditions from Na-Ge Zintl precursors such as Na_12_Ge_17_ (Figure 3c). As one example, the reaction of Na_12_Ge_17_ with n-dodecyltrimethylammonium chloride (DTAC) in the ionic liquid DTAC/AlCl_3_ can be understood as a heterogenous Hofmann-like elimination according to [63]:

8Na_12_Ge_17_ + 96[C_12_H_25_N(CH_3_)_3_]Cl → 24Ge_136_ + 96NaCl + 48H_2_ + 96(C_10_H_21_)CH=CH_2_ + 96N(CH_3_)_3_. (1)

The highly complex crystal structure of Na_12_Ge_17_, shown in Figure 3c [64], contains both [Ge_4_]^4−^ and [Ge_9_]^4−^ Zintl polyanionic clusters that are charge balanced by Na^+^ cations. In contrast to the formation of *allo*-Ge or Ge(*oP*32), the transformation of polyanionic clusters in Na_12_Ge_17_ into the 3D 4-bonded (4b) network of Ge atoms in the Ge_136_ framework requires not only new Ge-Ge bonds to be formed but also existing Ge–Ge bonds to be broken. Presumably, the Na atoms template the formation of the Ge_136_ framework as the [Ge_4_]^4−^ and [Ge_9_]^4−^ polyanions are oxidized and are then emptied from the clathrate framework cages as the reaction progresses. As with Li_7_Ge_12_, the reaction conditions can have a pronounced effect on the products. For example, spatially separating the precursor from the DTAC significantly improves the yield, reduces the fraction of amorphous by-products, and demonstrates the critical role of gaseous products of DTAC decomposition in the oxidation of the precursor and the heterogeneous nature of the reaction [63]. The generality of mild chemical oxidation of Zintl precursors for preparing silicon and germanium frameworks has now been demonstrated in the synthesis of a wide variety of clathrate phases [60,63,65,66,67,68,69], indicating these synthetic routes deserve continued investigation for the preparation of novel crystalline silicon and germanium modifications.

### 4.2. Thermal Decomposition of Zintl Precursors

**Figure 4:**
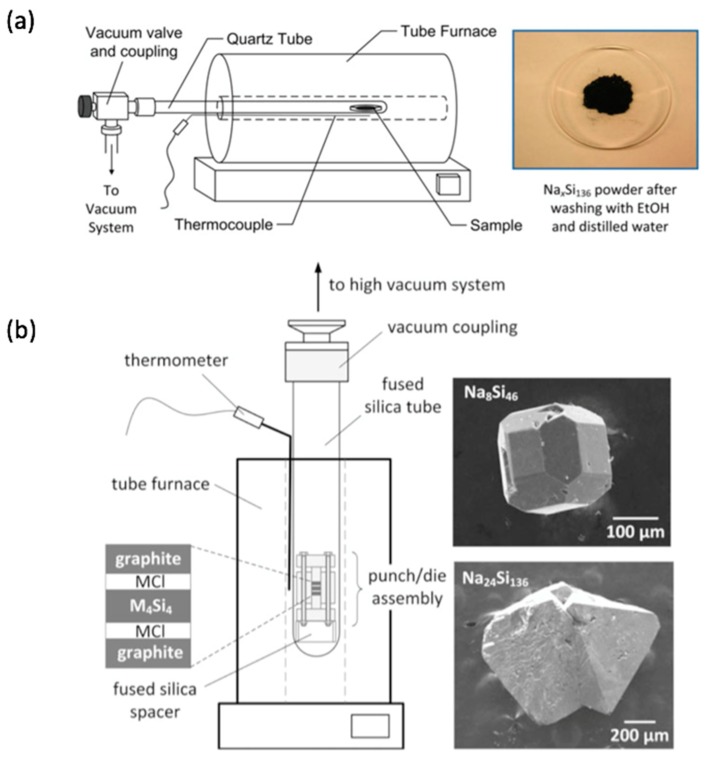
Two approaches to the synthesis of Na-Si clathrates (intermediates to Si clathrate modifications) by thermal decomposition of Na_4_Si_4_. The methods produce (**a**) microcrystalline and (**b**) single crystal products. In (**b**), the rate of thermal decomposition is controlled by maintaining a higher Na partial pressure (see [81]). Reproduced with permission from Nolas, G.S. (Copyright, 2014, Springer-Nature).

Historically, the synthesis of alternative 4b silicon and germanium frameworks from Zintl precursors, e.g., the clathrate frameworks (Figure 3c), actually preceded the report of *allo*-Si by many years. Cros and coworkers showed that the clathrate structures of silicon and germanium M_8_Tt_46_ or M_x_Tt_136_ (M = Na, K, Rb, or Cs; Tt = Si or Ge) are obtained upon the thermal decomposition (Figure 4a) of alkali metal silicon or germanium Zintl phases M_4_Tt_4_ containing [Tt_4_]^4−^ Zintl ions, e.g., Figure 3b [70,71]. Strictly speaking, these materials are not crystalline allotropes due to the presence of other elements in the structure, namely the guest atoms that occupy the cages formed by the Si or Ge framework. Nevertheless, the 4-coordinated bonding in these binary Si and Ge clathrates, which is similar to that in the corresponding *α*-phases, alluded to the possibility of obtaining expanded low-density silicon and germanium framework modifications if the guest atoms could be extracted [72]. With rare exceptions [73,74], the alkali metal atoms in clathrate-I M_8_Tt_46_ are not easily removed from their polyhedral cages. In contrast, Na atoms are readily removed from clathrate-II Na_x_Si_136_ upon heating under vacuum in the temperature range 350–450 °C [75]. Combining repeated heating under vacuum with density separation and/or reaction with iodine, the Na content can be reduced to ppm levels [76,77]. In this way, the new silicon allotrope Si(*cF*136) is obtained, whose widened electronic band gap [14,76] and significantly reduced thermal conductivity [22] can be attributed to the expanded clathrate crystal structure relative to *α*-Si. While in principle a pristine clathrate should be able to be obtained, in practice, some small alkali metal content (sodium) typically remains.

The preparation of crystalline allotropes by thermal decomposition of M-Si and M-Ge Zintl precursors is accompanied by several challenges. Often, more than one phase can be obtained in the reaction products. For example, thermal decomposition of Na_4_Si_4_ often results in a micro or nanocrystalline mixture of clathrate-I Na_8_Si_46_, clathrate-II Na_x_Si_136_, as well as *α*-Si and amorphous material [75]. Thermal decomposition of Na_4_Ge_4_ produces the zeolite-like Zintl phase Na_4_Ge_13_ [78,79] or clathrate-II Na_x_Ge_136_ depending on the decomposition conditions [80]. Recent improvements to thermal decomposition synthesis of these materials provide better control over the partial pressure of Na, improving the phase selectivity and access to phase pure macroscopic single crystals of Si-based clathrates (Figure 4b) [81,82]. Preparations of silicon clathrates using Zintl phases as precursors in metal flux synthesis are also being developed [83], while employing Na_4_Si_4_ as a precursor in conjunction with CsCl or KCl resulted in Si clathrate-I and II phases [84,85].

### 4.3. Reaction Pathways

All of the above synthetic approaches beg an important question—how do the isolated Si or Ge polyatomic anions in Zintl phase precursors transform into 3D 4-bonded frameworks upon oxidation? As with most solid-state reactions, in situ experimental data are sparse, and the detailed reaction mechanisms are not well understood. In one of the only in situ studies of the transformation of a tetrel-based Zintl phase into a tetrel-based framework, Hutchens et al. tracked the thermal decomposition of Na_4_Si_4_ to clathrate-I Na_8_Si_46_ using in situ synchrotron X-ray diffraction [86]. Despite the apparently disparate crystal structures of Na_4_Si_4_ (containing isolated [Si_4_]^4−^ clusters) and Na_8_Si_46_ (nominally containing a 3D [Si_46_]^8−^ 4-bonded framework), the authors found no evidence for any amorphous or crystalline intermediate phase. Rather, a large thermal expansion of the unit cell of Na_4_Si_4_ appears to enable a continuous transformation of one structure into another, accompanied by the simultaneous release of Na vapor from Na_4_Si_4_ [86]. Observed diffraction peaks common to both structures suggest a structural relationship between the two, though the exact relationship has not been elucidated.

The thermal behavior of Zintl precursors may be important in understanding the transformation that occurs in chemical synthesis routes as well. Future detailed in situ experiments, in particular those employing total scattering techniques [87], might yield important insight into the reaction pathways in the synthesis of silicon and germanium frameworks from Zintl precursors and how such routes might be extended to prepare other materials. Polyanion dynamics and cation diffusion pathways in the precursors and intermediates are also important but have thus far received relatively little attention [88,89,90,91].

### 4.4. Other Opportunities

The allotropes directly obtained from thermal or chemical treatment of Zintl phase precursors can also be further processed and transformed to yet other crystalline modifications. For example, m-allo-Ge transforms irreversibly to the 4H-Ge phase upon heating to approximately 200 °C [52,92]. The 4H-Ge phase then transforms to *α*-Ge near 400 °C [58]. Application of pressure to the low-density Ge(*cF*136) clathrate modification yielded the discovery of a new allotrope, Ge(*hR*8) [93].

The research described above provides a glimpse into the rich and complex free energy landscape for silicon and germanium elemental modifications and has revealed different ways that Zintl precursors can be utilized to access it. We conclude this section by noting that the chemical flexibility to prepare Zintl precursors having chemical substitutions on the tetrel sites provides even further opportunities to tune the properties of the resulting elemental modifications. For example, P-doping is possible on the Si sites of Na_4_Si_4_, which could in turn be used to dope a silicon modification derived from this precursor, e.g., Si(*cF*136) [42]. The ability to prepare Na_4_(Si_1−x_Ge_x_)_4_ precursors [43] has been utilized for the synthesis of silicon-germanium alloy clathrates via thermal decomposition [80,94], where the optical and transport properties can be tuned with the Si:Ge composition [95].

## 5. Nanoporous and Mesostructured Networks

**Figure 5:**
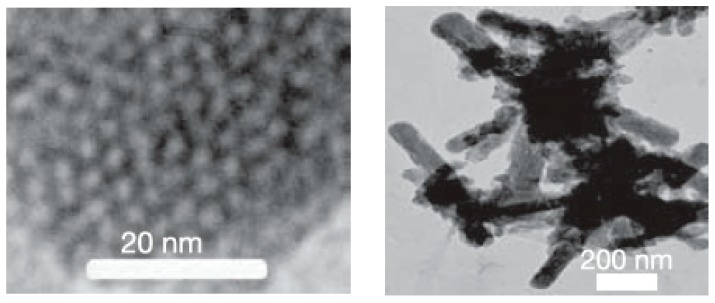
A view of the hexagonal array in a partly oxidized composite (left) and the particle shape of the product (right). Reprinted with permission from Sun, D. et al. (copyright, 2006, Nature).

**Figure 6:**
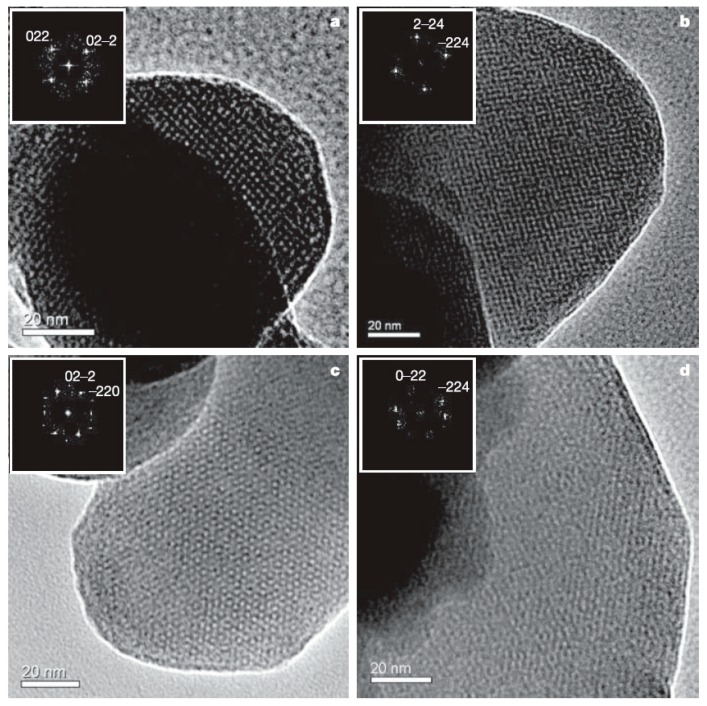
High-resolution TEM micrographs with fast Fourier transform (FFT) images as insets of mesoporous Ge prepared from Mg_2_Ge and GeCl_4_ taken along the [100] (**a**), the [110] (**b**), the [111] (**c**), and the [311] directions (**d**). Reproduced with permission from Armatas, G.S. et al. (copyright, 2006, Nature).

Mesostructured and mesoporous materials have been of interest for some time [96], but mesostructured silicon and germanium materials have only recently gained more attention with the development of effective methods of synthesis for non-oxides. Nano- or meso-porous Ge was initially prepared from Zintl phases for porous optical and electronic applications [97,98,99] and, more recently, for potential applications in Li ion batteries [100]. Some of these results were reviewed relatively recently [25]. Nanoporous Ge can be prepared from dissolving solid “K_2_Ge_9_” in ethylenediamine and mixing with cetyltriethylammonium bromide surfactant [97]. The dissolved [Ge_9_^2−^]_n_ polymer was oxidized with ferrocenium hexafluorophosphate to form a dark colored solid. Nanoporous Ge_1−x_Si_x_ was prepared from K_4_Ge_5_Si_4_ following the same procedure. The TEM image of Figure 5 shows the nanoscale order with a hexagonal array of pores for the product. Nanoporous Ge can also be prepared from the reaction of Mg_2_Ge with GeCl_4_ in the presence of the amphiphilic surfactant N-eicosane-N-methyl, N,N-dis(2-hydroxyethyl)ammonium bromide in formamide solution [98]. Figure 6 shows the high-resolution TEM and fast Fourier transform (FFT) as an inset, indicating the various orientations of cubic mesostructured germanium.

**Figure 7:**
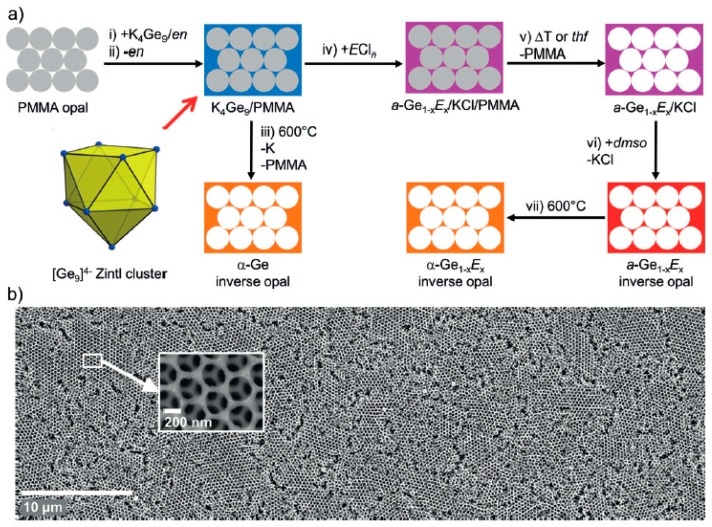
Schematic of the reaction to prepare mesoporous Ge by oxidation of Ge_9_^4−^ cluster (**a**) PMMA = polymethylmethacrylate, en = ethylenediamine. Scanning electron micrographs of Ge inverse opals with a magnified section indicated (**b**). Reproduced with permission Bentlohner, M.M. et al. (copyright, 2016, Angewandte Chemie International Edition).

[Ge_9_]^4−^ clusters have also been employed to make highly ordered porous frameworks [100,101]. Employing K_4_Ge_9_ as a soluble precursor in ethylenediamine and reacting with SiCl_4_, GeCl_4_, and PCl_3_ provides a Si and P-containing Ge mesoporous material, as demonstrated by powder X-ray diffraction, Raman spectroscopy, and energy-dispersive X-ray analysis. The main idea behind this work was to provide a homogeneous distribution of the Zintl phase K_4_Ge_9_ in a template mold and oxidize the [Ge_9_]^4−^ clusters via a metathesis reaction, as illustrated in Figure 7. The pores are templated by close-packed polymethylmethacrylate (PMMA) spheres, which yield Ge inverse opals upon removal. The authors demonstrated by Raman spectroscopy that the [Ge_9_]^4−^ remains intact as deposited, and the oxidation and removal of the salt and ethylenediamine result in amorphous Ge. Crystalline mesoporous Ge was obtained by heat treatment. Potential applications of such materials include hybrid solar cells and thin film anodes.

## 6. Nanoparticles and Other Nanostructures

Si and Ge semiconductor nanomaterials have useful applications in many areas of technology and biology [102,103,104,105]. In principle, the band gap of these materials can be controlled by size and surface modification. While decreasing the size will increase the band gap, the mobility of charge carriers is controlled through doping with either donors or acceptors. Employing Zintl phase precursors allows for significant advantages in obtaining new morphologies and unique properties. While there have been a number of reviews in employing Zintl phases for new clusters and nanomaterials, there are relatively few that focus on use of silicide and germanide precursors to make Si and Ge nanomaterials [28,33,106].

**Figure 8:**
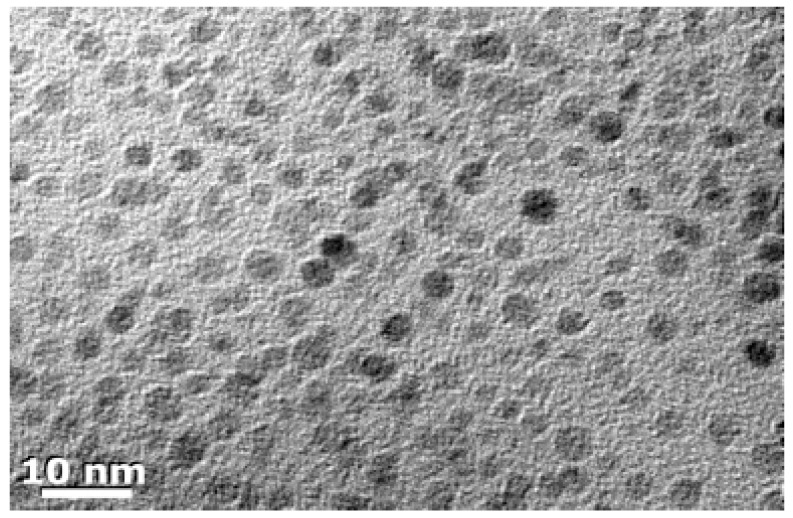
TEM micrograph of Si nanoparticles prepared from the reaction of Mg_2_Si with Br_2_ in 1,2-dimethoxyethane and are alkyl-/alkoxy-capped. Reproduced with permission from Pettigrew, A. et al. (copyright, 2003, American Chemical Society).

**Figure 9:**
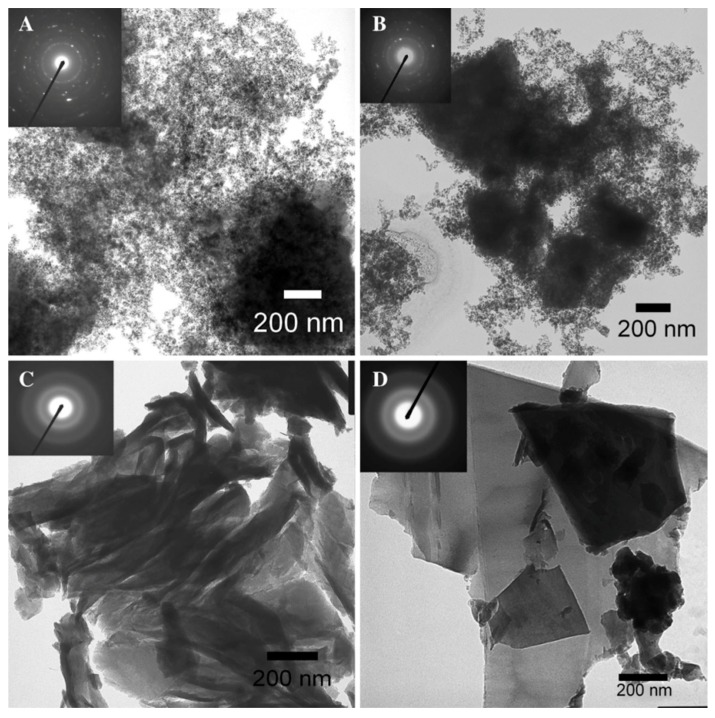
TEM micrographs [insets of selected area electron diffraction (SAED) for the samples] of Si nanoparticles prepared from the reaction of Na_4_Si_4_ with ethanol (**A**); 1-butanol (**B**); benzyl alcohol (**C**); 1-octanol (**D**). Reproduced from Oxidation pathways towards Si amorphous layers or nanocrystalline powders as Li-ion batteries anodes. Reproduced with permission from Annou, K. et al. (copyright, 2014, Materials for Renewable and Sustainable Energy).

Both Si and Ge nanomaterials can be prepared by multiple methods and chemical routes, but employment of Zintl phase precursors offers a facile and low temperature method to prepare them. Si and Ge nanoparticles (NPs) with controlled size from approximately 10 nm to 2 nm in diameter can be prepared from Zintl phases. Depending upon the route, the inorganic surfaces are terminated by either halogens or hydrogen. Metathesis is a well-known route to new compounds [107] and has been employed for making Si and Ge NPs in solution [108,109,110,111,112,113,114], as well as other nanomaterials [115]. Oxidation of metal silicides with bromine in solution yields highly crystalline hexagonally shaped nanocrystals of Si [116]. Figure 8 shows crystalline Si nanoparticles prepared via the oxidation reaction of Mg_2_Si and Br_2_ in solution. Some of the crystallites in the image have the expected hexagonal shape of diamond structured Si. Oxidation of Na_4_Si_4_ with alcohols shows that various morphologies of nanomaterials can be obtained [117]. The oxidation ability of the various alcohols provides either crystalline or amorphous materials, as shown in Figure 9. Short chain alkyls give rise to crystalline products, as shown in the selected area electron diffraction (SAED) patterns shown in the insets for each sample. The morphology of the final product was dictated by the reduction ability of the alcohol. The nanoparticles obtained from ethanol were 15 ± 4 nm in diameter, whereas those obtained from 1-butanol were smaller (11 ± 4 nm). Benzyl alcohol and 1-octanol gave large submicron sheets of amorphous materials. The effect of alcohol oxidation is also noted in metathesis reactions. For example, in the reaction of Mg_2_Si with SiI_4_ [115], the authors found that nanowires of Si could be prepared in quantitative yield by adding a small amount of ethanol. The probable reaction is the initial oxidation of Mg_2_Si to MgO and SiH_4_ + C_2_H_2_ and the heat released from this reaction drives the decomposition of SiI_4_ to Si and I_2_. The presence of I_2_ may contribute as a vapor transport agent, as the authors document that iodine gas is produced by the reaction. The authors also investigated other alcohols but found that branched alcohols did not have the same effect. This may be similar to results described above for alcohol oxidation; further research might provide a simple, effective way to obtain high yields of Si in particular morphologies. Similar to oxidation with alcohols, metal silicides and germanides can be transformed to nanomaterials by the application of ammonium halides [118], NH_4_X, to prepare Si and Ge nanomaterials [119,120,121,122], providing an easy route to high yields of Si and Ge nanomaterials.

**Figure 10:**
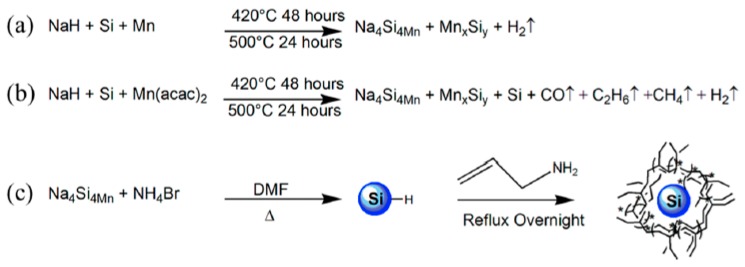
Reaction schemes for Mn doped Na_4_Si_4_ with Mn metal (**a**) Mn^II^ acetylacetonate (**b**) and the reaction scheme to form Mn doped Si nanoparticles (**c**). Reproduced from EPR and Structural Characterization of Water-Soluble Mn^2+^-Doped Si Nanoparticles. (copyright, 2017, The Journal of Physical Chemistry C).

**Figure 11:**
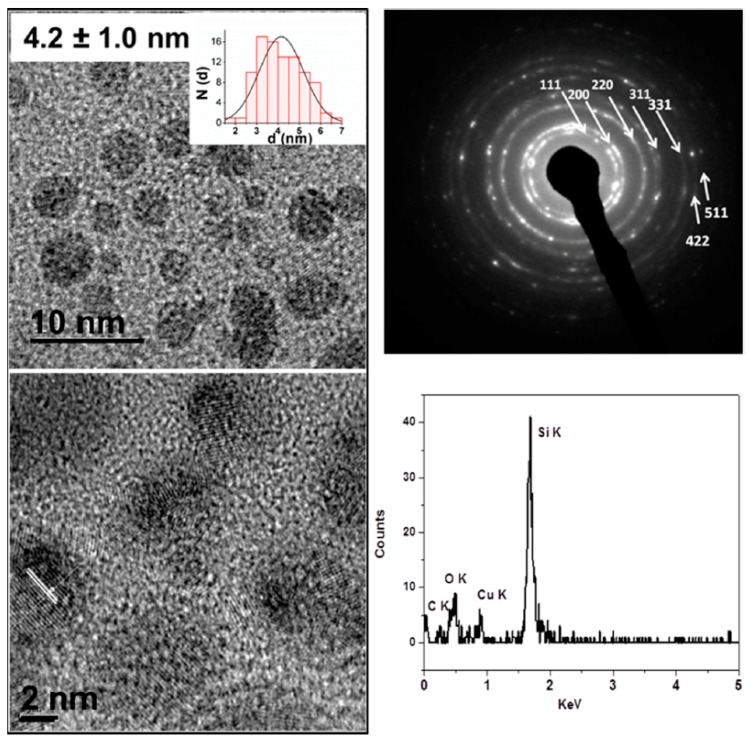
TEM images of Mn-doped Si nanoparticles with a histogram (top, left) and a high resolution TEM (bottom, left) with the (220) lattice plane indicated, Selected area electron diffraction (top, right) and energy dispersive X-ray spectroscopy (EDS) of the sample (bottom, right). The concentration of Mn was not sufficiently high to be observed in the EDS spectrum. Reproduced from EPR and Structural Characterization of Water-Soluble Mn^2+^-Doped Si Nanoparticles. (copyright, 2017, The Journal of Physical Chemistry C).

Transition metal doped and P doped Si NPs have been prepared by a reaction of metal or phosphorus doped Na_4_Si_4_ with NH_4_Br in solution [43,123,124,125,126,127]. Metal hydrides mixed with Si or Ge are reacted under an argon flow at low temperature to provide high purity A_4_Si_4_ and A_4_Ge_4_ (A = Na, K). To prepare doped Si and Ge nanomaterials, transition metal precursors such as metal acetylacetonates (or the element in the case of phosphorus [42]) are ball milled with Si according to Figure 10 [46] or Ge into a well dispersed fine powder and reacted with the metal hydride to form the metal silicide or germanide Zintl phase. These Zintl phases can then be employed with any of the reactions described above to make nanoparticles. The metal doped nanoparticles are characterized by TEM, X-ray powder diffraction, UV-Vis spectroscopy, photoluminescence, transient absorption spectroscopy, and electron paramagnetic resonance [125,126]. TEM analysis shows that the particles are crystalline, Figure 11. These nanoparticles have found applications as biocompatible and biodegradable multimodal agents [127,128,129,130]. In many cases, the yield of well capped crystalline NPs is low [105,121,131], but for other applications, such as Li ion batteries, amorphous products are better, and oxidation routes lead to different morphologies in quantitative yield [101,117].

Zintl phases containing group 14 elements in 2D layers have also been studied as precursors to unique layered materials that can be described as group 14 analogs of graphane, e.g., silicane and germane. Progress in this area was recently reviewed [106]. While we briefly highlight more recent work on oxidation of Zintl phases with alcohols, initial studies were conducted as early as the 1860s to prepare silicon sheets by the oxidation of CaSi_2_ (accompanied by removal of Ca from the structure) by HCl. The silicon layered material consists of a two-dimensional silicon backbone structure with stoichiometries of [Si_6_H_3_(OH)_3_]_n_ and [Si_6_H_6−x_(OH)_1−x_]_n_ (x < 1) depending on the reaction conditions [132,133]. These materials are considered to be puckered silicon layers with either terminal hydrogens and/or hydroxides [134]. Interestingly, they show direct bandgaps and photoluminescence.

**Figure 12:**
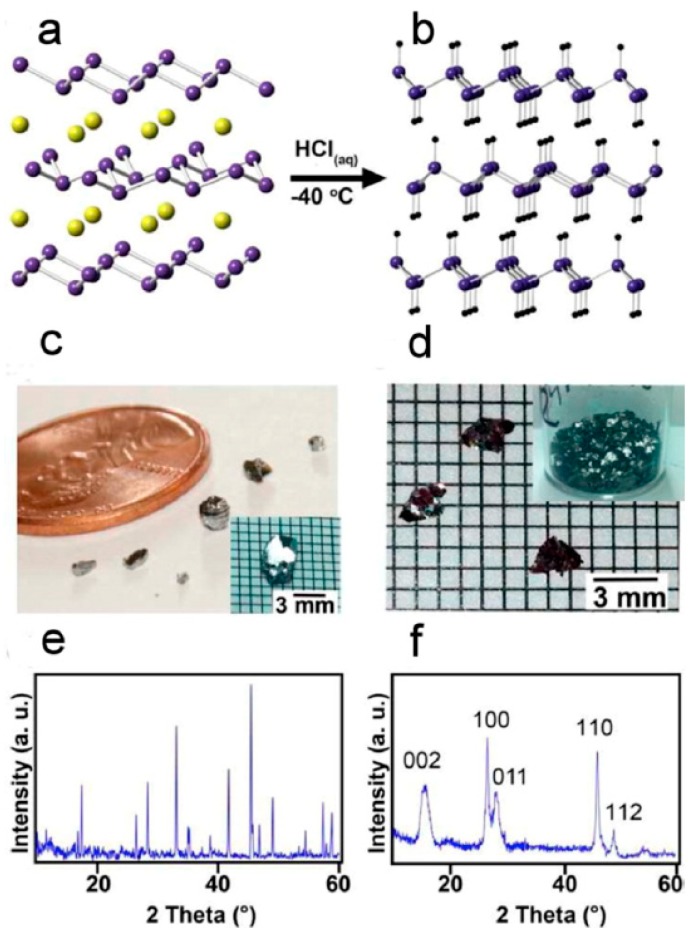
Illustration of the oxidation and deintercalation of CaGe_2_ (**a**) to GeH (**b**). Crystals of CaGe_2_ (**c**) and GeH (**d**) and powder X-ray diffraction of CaGe_2_ (**e**) and GeH (**f**). Reproduced with permission of Bianco, E. et al. (copyright, 2013, ACS Nano).

The above chemistry has also been extended to germanium, initially employing HCl [135]. More recently, this route has been scaled and demonstrated with large single crystals of CaGe_2_. Figure 12 shows the chemical reaction and the CaGe_2_ crystals with the product of GeH [136]. Since Zintl phases can be prepared with various amounts of the group 14 elements, this synthetic route has been expanded to include novel Ge_1−x_Sn_x_ 2D materials and more exotic group 14 materials [106]. The research on these novel 2D materials is still in its infancy, and the possibility of many combinations and chemical modifications is expected to stimulate more work on these materials, which may possess novel electronic and optical properties.

## 7. Outlook

The above examples illustrate the richness in novel silicon and germanium-based materials that can be prepared by thermal and chemical treatment of Zintl precursors containing these elements. A key feature of silicon and germanium-based Zintl phases that makes them particularly useful as precursors for synthesis of metastable materials is the presence of various reactive, covalently bonded anionic subunits, which can be oxidized to form 3D networks as well as a variety of nanostructures. Although in some cases the reaction pathways can be readily inferred by recognizing similarities between the features of the crystal structure of the precursor and the structure and bonding topology of the product, the concept of how polyanions in Zintl precursors transform to 4-bonded Si and Ge networks is still not well understood. In general, the product that will be produced by chemical or thermal treatment of a particular Zintl phase cannot easily be predicted a priori. This highlights the need for detailed in situ studies and additional theoretical work to better understand reaction pathways and mechanisms underlying the transformation of the Zintl precursors to crystalline modifications and other extended solids.

Inasmuch as chemical oxidation has proven a very effective approach to obtaining new materials from Zintl precursors, electrochemical oxidation also holds significant promise but as yet remains relatively underexplored. Work by Chandrasekharan and Sevov demonstrated that amorphous germanium films can be deposited anodically at room temperature by a relatively simple electrochemical process using K_4_Ge_9_ precursors dissolved in ethylenediamine [137]. Scherf et al. have demonstrated that *allo*-Ge can also be electrochemically prepared from Li_12_Ge_17_ [138]. On the other hand, macroscopic single crystals of clathrate-II Na_24_Si_136_ [139] and other silicon clathrates [140] are obtained by field assisted sintering of Na_4_Si_4_ at 600 °C under uniaxial pressure (so-called spark plasma sintering). Directed growth of Na_24_Si_136_ at the anode and accumulation of metallic Na at the cathode is suggestive of an influence of the electric current and solid-state electrochemical reaction. Such results hint at the potential of electrochemical reactions as applied to Zintl precursors, which would enable a greater variety of experimental parameters that can be used to direct the formation of specific products. Such approaches clearly deserve further study.

In closing, the research reviewed here has shown that Zintl precursors can be transformed into new materials via thermal treatment and metathesis reactions as well as solution, gaseous, and electrochemical oxidation. Perhaps other approaches, e.g., microwave synthesis, could be employed as well. While there has been progress in all areas, more rapid advances are expected once the various reaction pathways and mechanisms are better understood. We expect continued efforts will enable new and novel phases with interesting and perhaps useful properties while advancing the understanding of reaction mechanisms in solid-state chemistry in general.
